# Levels of adiponectin, a marker for PPAR-gamma activity, correlate with skin fibrosis in systemic sclerosis: potential utility as biomarker?

**DOI:** 10.1186/ar3827

**Published:** 2012-05-01

**Authors:** Katja Lakota, Jun Wei, Mary Carns, Monique Hinchcliff, Jungwha Lee, Michael L Whitfield, Snezna Sodin-Semrl, John Varga

**Affiliations:** 1 Department of Rheumatology, University Medical Centre Ljubljana, Vodnikova 62, Ljubljana 1000, Slovenia; 2 Department of Medicine, Feinberg School of Medicine, Northwestern University, East Huron Street 240, Chicago, 60611-2909 IL, USA; 3 Department of Preventive Medicine, Feinberg School of Medicine, Northwestern University, North Lake Shore Drive 680, Chicago, 60611-2909 IL, USA; 4 Department of Genetics, Dartmouth Medical School, Remsen 7400, Hanover, 03755 NH, USA

## Abstract

**Introduction:**

Progressive fibrosis in systemic sclerosis (SSc) is linked to aberrant transforming growth factor beta (TGF-beta) signaling. Peroxisome proliferator-activated receptor gamma (PPAR-gamma) blocks fibrogenic TGF-beta responses *in vitro *and *in vivo*. Reduced expression and function of PPAR-gamma in patients with SSc may contribute to progression of fibrosis. Here we evaluated the levels of adiponectin, a sensitive and specific index of PPAR-gamma activity, as a potential fibrogenic biomarker in SSc.

**Methods:**

Adiponectin levels were determined in the sera of 129 patients with SSc and 86 healthy controls, and serial determinations were performed in 27 patients. Levels of adiponectin mRNA in skin biopsies from SSc patients were assessed in an expression profiling microarray dataset. Regulation of adiponectin gene expression in explanted human subcutaneous preadipocytes and fibroblasts was examined by real-time quantitative PCR.

**Results:**

Patients with diffuse cutaneous SSc had reduced serum adiponectin levels. A significant inverse correlation between adiponectin levels and the modified Rodnan skin score was observed. In longitudinal studies changes in serum adiponectin levels were inversely correlated with changes in skin fibrosis. Skin biopsies from a subset of SSc patients showed reduced adiponectin mRNA expression which was inversely correlated with the skin score. An agonist ligand of PPAR-gamma potently induced adiponectin expression in explanted mesenchymal cells *in vitro*.

**Conclusions:**

Levels of adiponectin, reflecting PPAR-gamma activity, are correlated with skin fibrosis and might have potential utility as a biomarker in SSc.

## Introduction

Systemic sclerosis (SSc) is a multisystem disorder with protean clinical manifestations and substantial patient-to-patient heterogeneity [1]. Skin fibrosis characteristically shows rapid progression during early-stage disease but then reaches a plateau phase followed by slow regression. Transforming growth factor-beta (TGF-β) plays a key role in initiating and sustaining fibroblast activation and myofibroblast differentiation in SSc [2]. Microarray-based expression profiling of SSc skin biopsies shows an association that a 'TGF-β-activated gene signature' is associated with extensive skin involvement [3]. Multiple physiologic mechanisms regulate TGF-β signaling to prevent excessive tissue remodeling and fibrosis. One important endogenous anti-fibrotic defense mechanism involves the peroxisome proliferator-activated receptor-gamma (PPAR-γ) pathway, which blocks TGF-β responses [4].

The nuclear receptor PPAR-γ, initially identified in adipose tissue, plays key roles in regulation of adipogenesis, insulin sensitivity, and energy homeostasis [5]. In addition to being expressed in adipocytes, PPAR-γ is expressed in endothelial cells, vascular smooth muscle cells, macrophages, and fibroblasts [4,6]. Endogenous and diet-derived fatty acids and eicosanoids such as prostaglandin J_2 _(PGJ_2_) act as low-affinity natural PPAR-γ ligands, whereas the thiazolidenedione drugs are potent synthetic PPAR-γ agonists [7]. Recent studies identified an entirely novel function for PPAR-γ in regulation of matrix homeostasis [8]. Exposure of fibroblasts to pharmacological PPAR-γ ligands resulted in suppression of collagen synthesis, myofibroblast differentiation, and other TGF-β-induced fibrotic responses *in vitro *[4,9,10]. Moreover, treatment of mice with PPAR-γ agonists prevented and attenuated bleomycin-induced scleroderma *in vivo *[11]. The significance of the anti-fibrotic activity of PPAR-γ is highlighted by genetic targeting experiments that demonstrate constitutive collagen overexpression and excessive fibrogenesis in PPAR-γ-null fibroblasts *in vitro *and *in vivo *[12]. Importantly, PPAR-γ expression and activity are reduced in SSc skin biopsies and explanted fibroblasts and are inversely correlated with fibrogenic markers in the lesional skin [13,14]. These observations suggest that impaired PPAR-γ expression or function underlies unopposed fibroblast activation and progression of fibrosis in SSc. Assessing PPAR-γ expression or activity therefore might be a novel approach for assessing fibrogenic activity in SSc.

Adiponectin is a 244-amino acid hormone secreted from white adipose tissue that regulates insulin sensitivity and energy balance. The adiponectin gene is located on chromosome 3q27, a susceptibility locus for diabetes and metabolic disorders. Adiponectin transcription is tightly regulated by PPAR-γ through direct binding to conserved cis-acting regulatory DNA elements. Circulating adiponectin is decreased in obesity and the metabolic syndrome, and levels are increased in mice and in humans after treatment with PPAR-γ agonist agents [15]. Since serum adiponectin levels faithfully mirror PPAR-γ activity, adiponectin is now increasingly used as a biomarker of efficacy for PPAR-γ therapy [15]. In view of the PPAR-γ defect seen in SSc, we hypothesized that circulating adiponectin might be reduced in some patients with SSc. We therefore determined adiponectin levels in 129 patients with well-characterized SSc and correlated levels with clinical and laboratory features of disease. The results reveal significantly reduced adiponectin levels in diffuse cutaneous SSc (dcSSc) patients with early-stage disease, inverse correlation with the extent of skin fibrosis, and rising levels that parallel improved skin scores over time.

## Materials and methods

### Patients

Written informed consents, approved by the institutional review board of Northwestern University were acquired and serum samples were obtained from 129 patients with SSc - 50 with dcSSc and 79 with limited cutaneous SSc (lcSSc) - evaluated at the Northwestern Scleroderma Program between February 2009 and April 2010. In an additional cohort of 27 patients with dcSSc, two serum samples separated by more than 10 months were obtained and analyzed. Clinical and laboratory information obtained at the time of serum sampling included age, gender, ethnicity, smoking history, body mass index (BMI), disease duration (interval between first SSc-related non-Raynaud event and blood sampling), modified Rodnan skin score (MRSS) (0 to 51), forced vital capacity (FVC), and carbon monoxide diffusion capacity (DlCO) as a percentage of predicted high-resolution computerized tomography of the chest (Table 1 and Additional file 1). Screening for pulmonary hypertension (PH) was performed by Echo/Doppler or by right heart catheterization and provisionally defined as estimated pulmonary artery systolic pressure of at least 40 mm Hg or mean pulmonary artery pressure of at least 25 mm Hg, respectively. Anti-nuclear autoantibodies were detected by indirect immunofluorescence, and antibodies against topoisomerase-1, centromere, and RNA polymerase III were assayed. Control serum samples were collected from 86 healthy volunteers (Table 1).

**Table 1:** Demographic data

	Controls (*n *= 86)	Patients with SSc (*n *= 129)	Patients with dcSSc (*n *= 50)	Patients with lcSSc (*n *= 79)
Age in years, median (range)	43 (19-64)	53 (21-82)	51 (21-65)	55 (21-82)
Females, percentage	30	85	78	88
Ethnicity				
Caucasian	100%	71%	68%	75%
African-American		14%	14%	13%
Other		15%	18%	12%
Disease duration	N/A			
0-18 months		19%	36%	9%
19-36 months		10%	12%	9%
> 36 months		71%	52%	82%
MRSS from 0 to 51, median (range)	N/A	6 (2-45)	16 (2-45)	4 (2-25)
BMI in kg/m^2^, median (range)	N/A	25 (14-48)	25 (18-39)	25 (14-48)

BMI, body mass index; dcSSc, diffuse cutaneous systemic sclerosis; lcSSc, limited cutaneous systemic sclerosis; MRSS, modified Rodnan skin score; N/A, not applicable; SSc, systemic sclerosis.

### Determination of serum adiponectin levels

Serum samples were frozen at -80°C until assayed. Adiponectin levels were measured by using a multiplex assay kit (EMD Millipore, Billerica, MA, USA) in accordance with the instructions of the manufacturer. Briefly, serum samples (1:100 dilution) and standards were added to the wells, along with sonicated beads. After incubation and washing of the wells, antibodies were added, followed by streptavidin-phycoerythrin. Wells were then washed, and measurements on a Luminex 100 platform (Luminex Corporation, Austin, TX, USA) were performed. The assay detects all three (low-, medium-, and high-molecular weight) forms of adiponectin.

### Regulation of adiponectin gene expression

Human subcutaneous preadipocytes (Zen-Bio, Inc., Research Triangle Park, NC, USA) and neonatal foreskin fibroblasts were maintained at 37°C in an atmosphere of 5% CO_2 _in preadipocyte medium (PM) (Zen-Bio, Inc.) or Dulbecco's modified Eagle's medium supplemented with 10% fetal bovine serum, 1% vitamins, 1% penicillin/streptomycin, and 2 mM L-glutamine (all from BioWhittaker, Walkersville, MD, USA). Cells were studied between passages 4 and 8 at early confluence. Cultures were incubated in the presence or absence of pioglitazone (10 μM). After 48 hours of incubation, cultures were harvested, and mRNA levels were examined by real-time quantitative polymerase chain reaction (qPCR) by using primers for adiponectin (forward, 5'-TATCCCCAACATGCCCATTCG-3'; reverse, 5'-TAGGCAAAGTAGTACAGCCCA-3') and glyceraldehyde-3-phosphate dehydrogenase (GAPDH) (forward, 5'-CATGAGAAGTATGACAACAGCCT-3'; reverse, 5'-AGTCCTTCCACGATACCAAAGT-3').

### Adiponectin gene expression in skin biopsies

The generation and analysis of the genome-wide expression microarray dataset using skin biopsies from patients with SSc and healthy controls have been described (Gene Expression Omnibus accession number GSE9285) [16]. The expression levels of adiponectin mRNA in each skin biopsy were extracted from this dataset and centered on its mean value across all arrays.

### Statistical analysis

Mann-Whitney *U *tests or Kruskal-Wallis tests were used to compare adiponectin levels. The correlation between adiponectin levels and various values were analyzed by Spearman rank correlation test. Owing to non-normal distribution of the data, summary statistics are expressed as medians and interquartile ranges (IQRs). Data were analyzed by using SPSS Statistics 19 (SPSS Inc., Chicago, IL, USA). A *P *value of less than 0.05 was considered statistically significant.

## Results and Discussion

**Figure 1:**
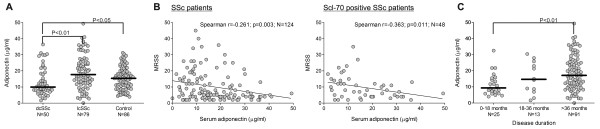
**Serum adiponectin levels in systemic sclerosis (SSc)**. Sera from 129 patients with limited (lcSSc) or diffuse (dcSSc) cutaneous SSc and 86 controls were analyzed by multiplex assays. **(a) **Horizontal bars indicate median values in each group. **(b) **Correlation between adiponectin levels and modified Rodnan skin score (MRSS) in all patients with SSc (left panel) and patients with Scl-70-positive SSc (right panel). The solid line represents the linear regression line. **(c) **Adiponectin levels in patients with SSc according to disease duration (defined as duration from first non-Raynaud manifestation). Horizontal bars indicate median values.

As expected, serum adiponectin levels in both control subjects and SSc patients were positively correlated with age and negatively correlated with BMI. Moreover, adiponectin levels were significantly lower in males than females, as reported previously [17]. The median levels of adiponectin were 14.7 μg/mL (IQR 15.4) among patients with SSc and 15.3 μg/mL (IQR 11.2) in healthy controls (*P *= 0.49). While there were no significant differences in adiponectin levels between patients and controls when adjusted for differences in age, gender, and ethnicity, patients with dcSSc (*n *= 50) had significantly lower adiponectin levels (median 10.0 μg/mL, IQR 11.3) than those with lcSSc (*n *= 79, median 18.3 μg/mL, IQR 13.1, *P *< 0.001) (Figure 1a). Further analyses revealed significant differences in adiponectin levels between SSc patients with or without anti-centromere antibody: median 21.5 μg/mL (IQR 20.3) versus 13.3 μg/mL (IQR 14.8) (*P *= 0.015), respectively. When grouped according to disease duration, patients with disease in a relatively early stage (arbitrarily defined as fewer than 18 months from the first non-Raynaud manifestation, *n *= 25) had low adiponectin levels (median 9.6 μg/mL, IQR 8.5) in comparison with patients with disease in the late stage (defined as more than 36 months, *n *= 91) (median 17.1 μg/mL, IQR 15.3, *P *= 0.004) (Figure 1c). The lower levels of adiponectin in the early stage of disease, when progression of skin fibrosis is thought to be the most active, might reflect reduced tissue PPAR-γ activity. The differences in adiponectin levels were significant even when adjusted for age, gender, ethnicity, and BMI (*P *< 0.001).

The difference in adiponectin levels between patients with lcSSc and those with dcSSc suggested that adiponectin, as a marker of PPAR-γ signaling in the skin, might be correlated with skin fibrosis. Indeed, we demonstrated a weak but statistically significant inverse correlation between adiponectin levels and the extent of skin fibrosis (r = -0.261, *n *= 124, *P *= 0.003) (Figure 1b). This correlation persisted even when corrected for age, gender, ethnicity, and BMI. A more robust correlation between MRSS and adiponectin levels was seen in Scl-70-positive patients, suggesting the association of reduced PPAR-γ activity with active skin disease (Figure 1b). In contrast to the skin score, neither FVC nor DlCO or radiologic evidence of pulmonary fibrosis could be demonstrated to have a significant correlation with serum adiponectin levels (Additional files 1 and 2). The correlation of serum adiponectin levels with skin but not lung fibrosis points to potential differences in the pathomechanisms underlying fibrosis in these organs, and PPAR-γ has a more prominent role in skin fibrosis than in lung fibrosis.

**Figure 2:**
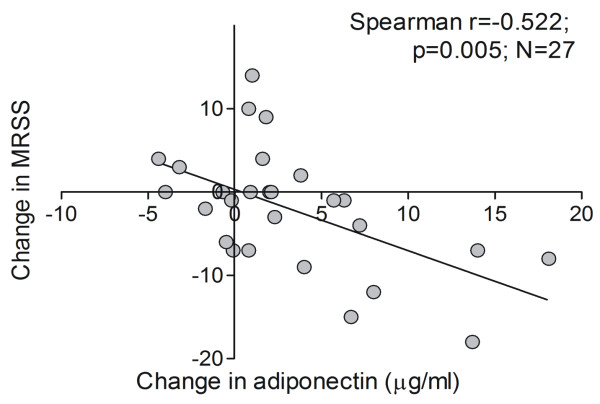
**Changes in adiponectin levels over time**. Serum adiponectin levels were determined in 27 patients with diffuse cutaneous systemic sclerosis at two time points separated by more than 10 months. Changes in adiponectin levels are correlated with contemporaneous changes of modified Rodnan skin score (MRSS).

To examine changes in adiponectin levels over time, serial serum samples that were from the same patients and that were separated by more than 10 months were examined in 27 patients. Eighteen received mycophenolate mofetil alone or in combination with methotrexate, and seven received no specific disease-modifying therapy ([18] and Additional file 3). The results of the longitudinal analysis indicated that changes in adiponectin levels over time were inversely correlated with changes in skin score during the same interval (r = -0.522, *n *= 27, *P *= 0.005) (Figure 2). There were no apparent differences in changes in skin score or adiponectin levels associated with specific treatments.

**Figure 3:**
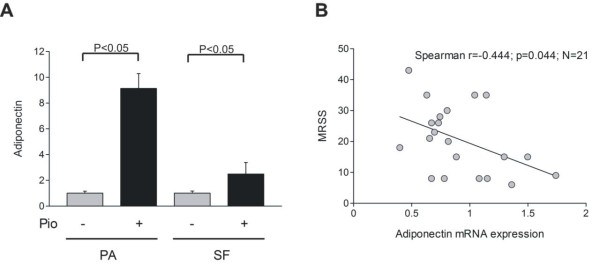
**Adiponectin mRNA regulation**. **(a) **Confluent human skin fibroblasts (SFs) or subcutaneous preadipocytes (PAs) were incubated with 10 μM pioglitazone (Pio) for 48 hours, and RNA was examined by real-time quantitative polymerase chain reaction. The results, normalized with glyceraldehyde-3-phosphate dehydrogenase mRNA levels, represent the mean ± standard deviation of triplicate determinations. **(b) **Adiponectin mRNA levels in systemic sclerosis skin biopsies. The data were extracted from published genome-wide expression microarray datasets [16]. Levels were correlated with modified Rodnan skin score (MRSS).

The adiponectin gene contains a conserved peroxisome proliferator responsive element (PPRE) that is targeted by activated PPAR-γ, resulting in ligand-dependent transcription [19]. To examine adiponectin regulation in mesenchymal cells, growth-arrested confluent normal human preadipocytes or dermal fibroblasts were incubated with the PPAR-γ ligand pioglitazone for 48 hours. The results of real-time qPCR analysis indicated increases of greater than threefold and greater than ninefold in adiponectin mRNA levels of pioglitazone-treated fibroblasts and preadipocytes, respectively (Figure 3a). Reduced levels of circulating adiponectin in patients with early-stage dcSSc suggested attenuated PPAR-γ signaling during active fibrogenesis. To explore this possibility, we examined adiponectin mRNA expression in lesional SSc skin biopsies. For this purpose, a published genome-wide expression profiling dataset comprising biopsies of the lesional (clinically affected) skin from 15 patients with early dcSSc and six patients with lcSSc was queried [16]. A significant correlation between adiponectin mRNA expression in the skin and MRSS was observed in the individual biopsies (r = -0.44, *n *= 21, *P *= 0.044) (Figure 3b).

Taken together, the present results provide support for the involvement of PPAR-γ in modulation of skin fibrogenesis in SSc and suggest that reduced adiponectin levels might reflect reduced PPAR-γ activity associated with active or progressive skin fibrosis. Defective PPAR-γ activity in SSc contributes to unopposed fibroblast activation and is implicated in pathogenesis [14]. Adiponectin production is tightly regulated by PPAR-γ, and its levels in the serum represent a robust biomarker of PPAR-γ activity [15]. We found significantly lower serum adiponectin levels in patients with dcSSc than in those with lcSSc, and levels were lowest in the early stage of disease, when fibrogenesis is presumed to be the most active. A gradual rise in the levels of adiponectin over time might parallel spontaneous or treatment-associated skin regression and may signal attenuation of fibrogenesis. The present studies did not compare adiponectin levels in SSc with those with other autoimune diseases. However, studies have shown that rheumatoid arthritis, systemic lupus erythematosus, and other immune and inflammatory disorders are generally associated with elevated serum adiponectin [20-24]. Thus, it is noteworthy that, in contrast, SSc is not associated with elevated adiponection, despite the chronic inflammation that is thought to be a hallmark of this disease [25].

The levels of adiponectin in the circulation reflect PPAR-γ signaling activity in mesenchymal cells [15]. Thus, reduced adiponectin levels and adiponectin gene expression in lesional tissue in SSc indicate reduced adiponectin production that results from the impaired PPAR-γ activity. Adiponectin directly suppresses fibroblast migration and myofibroblast differentiation and blocks TGF-β-induced fibrotic responses, including collagen synthesis [26]. Moreover, adiponectin-null mice show exaggerated fibrogenesis in the heart, liver, and kidneys, suggesting a potential anti-fibrotic role [27]. Whether adiponectin is simply a marker for PPAR-γ signaling or, in fact, mediates its anti-fibrotic activities is currently unknown.

## Conclusions

In summary, this cross-sectional analysis indicates that the levels of the PPAR-γ-regulated adiponectin are reduced in early-stage dcSSc and are inversely correlated with the skin score. Moreover, rising levels of adiponectin over time correlate with improvement in skin score. Reduced adiponectin reflecting impaired PPAR-γ activity might signify unopposed fibroblast activation and serve as a biomarker for ongoing fibrogenesis. Two recent studies with fewer patients reported reduced adiponectin levels in Japanese patients with SSc [20,28]. These findings are consistent with our present results with a significantly larger patient cohort of a different ethnic background. Taken together, the results provide a compelling case for a role of PPAR-γ activity in skin fibrosis in SSc and support the prospective evaluation of adiponectin as a biomarker of fibrogenic activity.

## Abbreviations

BMI: body mass index; dcSSc: diffuse cutaneous systemic sclerosis; DlCO: carbon monoxide diffusion capacity; FVC: forced vital capacity; IQR: interquartile range; lcSSc: limited cutaneous systemic sclerosis; MRSS: modified Rodnan skin score; PH: pulmonary hypertension; PPAR-γ: peroxisome proliferator-activated receptor gamma; qPCR: quantitative polymerase chain reaction; SSc: systemic sclerosis; TGF-β: transforming growth factor-beta.

## Competing interests

The authors declare that they have no competing interests.

## Authors' contributions

JV helped to conceive the study and draft the manuscript. KL carried out serum assays and statistical analyses and helped to conceive the study and draft the manuscript. JL participated in statistical analysis of data and evaluation of the manuscript. JW carried out cell culture experiments and mRNA analysis. MH and MC collected disease samples and clinical data. MLW carried out genome-wide expression analyses and critical evaluation of the manuscript. SSS provided healthy control samples with data and helped to write the manuscript. All authors read and approved the final manuscript.

## Supplementary Material

Additional file 1 **Patient adiponectin levels according to clinical and laboratory features**. Adiponectin levels evaluated statistically in different clinical and laboratory features. Significance was tested by Mann Whitney (MW) test or Kruskal Wallis (KW) test. NS = not significant. Pulmonary hypertension defined as estimated pulmonary artery systolic pressure > 40 mmHg (Echo/Doppler), or mean pulmonary artery pressure by right heart catheterization > 25 mmHg. Click here for file

Additional file 2 **Median levels of clinical/laboratory scores in patients within different adiponectin quartiles and their correlation coefficients**. Correlation coefficients were determined using Spearman rank correlation test. PA = mean pulmonary artery pressure based on right heart catheterization. BNP = brain natriuretic peptide Interquartile ranges are in parentheses. NS = not significant. Click here for file

Additional file 3 **Levels of adiponectin and MRSS in the longitudinal study**. Levels of adiponectin at 2 time points (visit 1: MRSS1, adiponectin 1; visit 2: MRSS 2, adiponectin 2) with an indication of time laps in between the two visits. MRSS = modified Rodnan skin score. Click here for file
